# Outcome of COVID-19 in Solid Organ Malignancies: Experience From a Tertiary Cancer Center in Eastern India

**DOI:** 10.1200/GO.21.00139

**Published:** 2021-09-10

**Authors:** Somnath Roy, Joydeep Ghosh, Sandip Ganguly, Debapriya Mondal, Deepak Dabkara, Soumyadip Chatterji, Bivas Biswas

**Affiliations:** Department of Medical Oncology, Tata Medical Center, Kolkata, India

## Abstract

**PURPOSE:**

The COVID-19 pandemic has imposed a unique challenge to oncology patients. Outcome data on COVID-19 in patients with cancer from the Indian subcontinent are scarce in the literature. We aimed to evaluate the outcome of patients with COVID-19 on active systemic anticancer therapy.

**MATERIALS AND METHODS:**

This is a retrospective study of patients with solid organ malignancies undergoing systemic therapy with a diagnosis of COVID-19 between March 2020 and February 2021. COVID-19 was diagnosed if a reverse transcriptase polymerase chain reaction assay from oropharyngeal or nasopharyngeal swab was positive for severe acute respiratory syndrome coronavirus 2. The objectives were to evaluate the outcome of COVID-19 and factors predicting the outcome.

**RESULTS:**

A total of 145 patients were included with a median age of 58 years (range, 20-81 years). Treatment was curative in 60 (42%) patients. Of all symptomatic cases (n = 88, 61%), 50 had mild, 27 had moderate and 19 had severe COVID-19–related symptoms as per WHO criteria. Fifty (34%) patients required hospitalization with a median duration of hospital stay of 12 days (range, 4-25 days); five patients required intensive care unit admission. The rest were treated with home isolation and did not require further hospitalization. Twenty-two (15%) patients died, and the risk of death was significantly associated with severity of symptoms (odds ratio, 91.3; 95% CI, 9.1 to 919.5, *P* = .0001) but not with any other clinical factors. Drug holiday was given to 63 (44%) patients with a median duration of 25 days (range, 7-88 days). The median duration to reverse transcriptase polymerase chain reaction–negative was 16 days (range, 7-62 days).

**CONCLUSION:**

COVD-19–related death rate was 15% among patients with solid organ malignancies. The severity of the symptoms was related to mortality. The majority of patients with mild symptoms were treated at home isolation.

## INTRODUCTION

The novel coronavirus (SARS-nCoV-2) has affected the entire globe, with different countries in various stages of the pandemic and so far 119,220,681 peoples affected globally with 2,642,826 confirmed deaths as per WHO report.^1^ The Indian Government declared a nationwide lockdown on March 24, 2020, in view of the rapid spread of this virus.^2^

**CONTEXT**

**Key Objective**
Oncology patients are more to severe COVID-19 owing to their depressed immunity, malnutrition, and chemotherapy-related myelosuppression, etc. Fear had griped the oncology community to interrupt or modify the cancer treatment. Many publications showed increased COVID-19–related morality in patients with cancer compared with healthy individuals and hence, many guidelines published to customize the cancer treatment in a risk-based approach. There is only one publication so far about the COVID-19–related outcome in Indian oncology patients.
**Knowledge Generated**
Here, we have evaluated the same outcome in our solid organ patients undergoing active anticancer therapy. Case fatality rate (CFR) was less compared with Western literature. CFR was directly related to severity of COVID-19–related symptoms. Patients with lung cancer had the highest CFR. Majority of mild symptomatic patients were treated at home.
**Relevance**
Our study will boost the morale of oncology community that active anticancer treatment can be continued for oncology patients affected with COVID-19 and that CFR is less.

Although the case fatality rate (CFR) in India was lower than the available data from European countries and North America, it significantly misbalanced the entire health care delivery system in our country.^3^ The UK Coronavirus Cancer Monitoring Project (UKCCMP) and the COVID-19 and Cancer Consortium (CCC19) consortium database confirmed the higher risk of COVID-19–related mortality and morbidity among patients with cancer undergoing active systemic therapy compared with the general population.^4,5^

Mehta et al identified that CFR was high among patients with cancer because of older age of diagnosis with coexisting medical comorbidities, malnutrition, and cancer cachexia, increased susceptibility to infection because of myelosuppressive effects of chemotherapy, hematologic malignancy with underlying immunosuppression, and cytokine storm secondary to hyperinflammation leading to pulmonary damage etc. This study also showed that the CFR was almost double (28% *v* 14%) among patients with cancer compared with the matched noncancer population with COVID-19.^6^

Another case-control study from United States in August 2020 demonstrated that out of 16,570 patients diagnosed with COVID-19 infection, 1,890 (11%) patients were suffering from at least one common cancer, and among those suffering from hematologic malignancies, and those with lung cancer were at significantly increased risk for COVID-19 infection.^7^ However, there are limited data from our country to differentiate the behavior and outcomes of COVID-19–positive cases undergoing active systemic treatment.^8,9^

We aimed to report the real-world experience from a single institute in the eastern part of India regarding the demographic characteristics, treatment details, and outcome of COVID-19–positive cases on active systemic therapy in various solid tumors.

## MATERIALS AND METHODS

This is a retrospective observational study of all solid organ malignancy patients with COVID-19 from March 2020 to February 2021 at our department. In view of the retrospective nature of the study, waiver of consent was received from the institutional review board as per institutional policy (IEC Protocol Waiver No - EC/WV/TMC/08/21).

All patients age ≥ 18 years irrespective of stage and intent of therapy with laboratory-confirmed COVID-19 positivity were considered eligible for this study. Patients who received definitive surgery only, concurrent chemoradiation, and concomitant hematologic malignancies were excluded from the study. As per institutional protocol, COVID-19 was diagnosed if a reverse transcriptase polymerase chain reaction (RT-PCR; by TRU PCR kit, Blackbio, India and Rotor Gene-Q, Qiagen, Germany) assay from oropharyngeal or nasopharyngeal swab was positive for severe acute respiratory syndrome coronavirus 2 (SARS-CoV-2). As a departmental policy, RT-PCR for severe acute respiratory syndrome coronavirus 2 was only performed in symptomatic patients, asymptomatic patients needing any elective procedure, and patients who required admission for systemic therapy–related toxicities.

Repeat testing was conducted after initial RT-PCR positivity for evaluation of recovery as per government guidelines of treating patients with cancer as appropriate. All supportive care data like use of steroid, remdesivir, anticoagulant, and intensive care unit (ICU) were corrected and analyzed.

### Data Collection

Data were collected from electronic medical records from our hospital. Demographic details, including age, sex, Eastern Cooperative Oncology Group performance status, comorbidity, primary site and stage of malignancy, the intent of therapy, severity of COVID-19 symptoms, neutrophil-to-lymphocyte ratio (NLR), and platelet-to-lymphocyte ratio (PLR), change of anticancer treatment strategy because of COVID-19 infection, hospital admission and ICU support, COVID-19–related treatment details, home isolation, and recovery from COVID-19 with test result negativity (wherever applicable), were retrieved. The severity of COVID-19 was categorized as per WHO guidelines into mild, moderate, and severe.

### End Points

The primary end point was the outcome in terms of recovery or death from COVID-19 cases undergoing active systemic therapy on solid organ malignancies.

### Statistical Analysis

Descriptive statistics, tables, and charts were used to analyze the demographic, clinical, and treatment-related variables. To compare binomial categorical variables, Pearson's chi-squared test and odds ratio were used. Various factors influencing the outcome were evaluated by binomial logistic regression analysis. Statistical analysis was done using STATA/SE11.0 (Stata Corp LP, TX).

## RESULTS

A total of 145 patients were included in this study with a median age of 58 years (range, 20-81 years) and a male:female ratio of 75:43. The demographic characteristics are mentioned in Table 1. There were 43 (29.7%) chronic smokers, 82 (56.5%) nonsmokers, and 20 (13.8%) ex-smokers. The most common comorbidities were hypertension in 32 (22%), diabetes in 39 (26%), and coronary artery disease in 12 (8%) patients. The common primary sites of cancer were lung cancer in 37 (26%), breast cancer in 35 (24%), gastrointestinal cancer in 23 (15%), genitourinary cancer in 23 (15%), and gynecologic cancer in 16 (11%) patients. Ninety (62%) patients had advanced disease. Treatment status was active treatment in 112 (77%), follow-up in 14 (10%), and under evaluation in 19 (13%) patients. The anticancer treatment types were myelosuppressive cytotoxic chemotherapy in 57 patients, nonmyelosuppressive chemotherapy in 41 patients, immunotherapy in five patients, and oral tyrosine kinase inhibitors in nine patients (Table 1).We continued the same treatment regimen and protocol for our patients but a drug holiday was given in 63 (44%) patients and the regimen was changed to oral monotherapy for only one patient. The median duration of drug holiday was 25 days (range, 7-88 days).

**TABLE 1:**
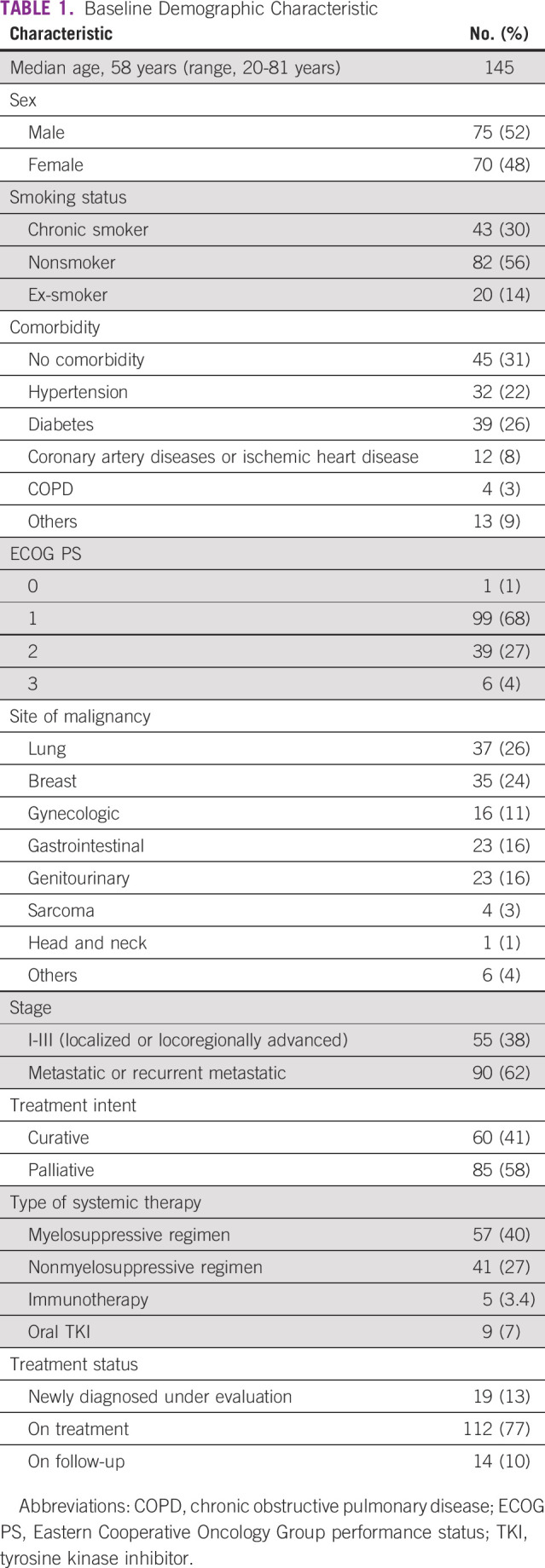
Baseline Demographic Characteristic

At presentation, 88 (61%) patients were symptomatic for COVID-19 and 57 (39%) were asymptomatic carriers (detected on screening for elective procedures). The median duration of symptoms at presentation was 6 days (range, 2-20 days). Around 60% of our patients were symptomatic positive, and the severity of symptoms was mild in 50 (34%), moderate in 27 (18%), and severe in 19 (13%) patients as per WHO guidelines of the severity of COVID-19–related symptoms. Fifty (34%) patients required hospital admission for supportive care, with a median duration of hospital stay of 12 days (range, 4-25 days) and out of them, five patients were shifted to ICU. Among admitted patients, dexamethasone was given in 24 (16%), remdesivir was used in 8 (5%), and anticoagulant in 24 (16%) patients. The median value of NLR was 6.2 (range, 1.1-25), whereas the median value of PLR was 487 (range, 7-494), which were not significantly associated with mortality. Among those who recovered from COVID-19, cancer progression was seen in six patients only. Details of treatment offered for the management of COVID-19 are briefly outlined in Table 2.

**TABLE 2:**
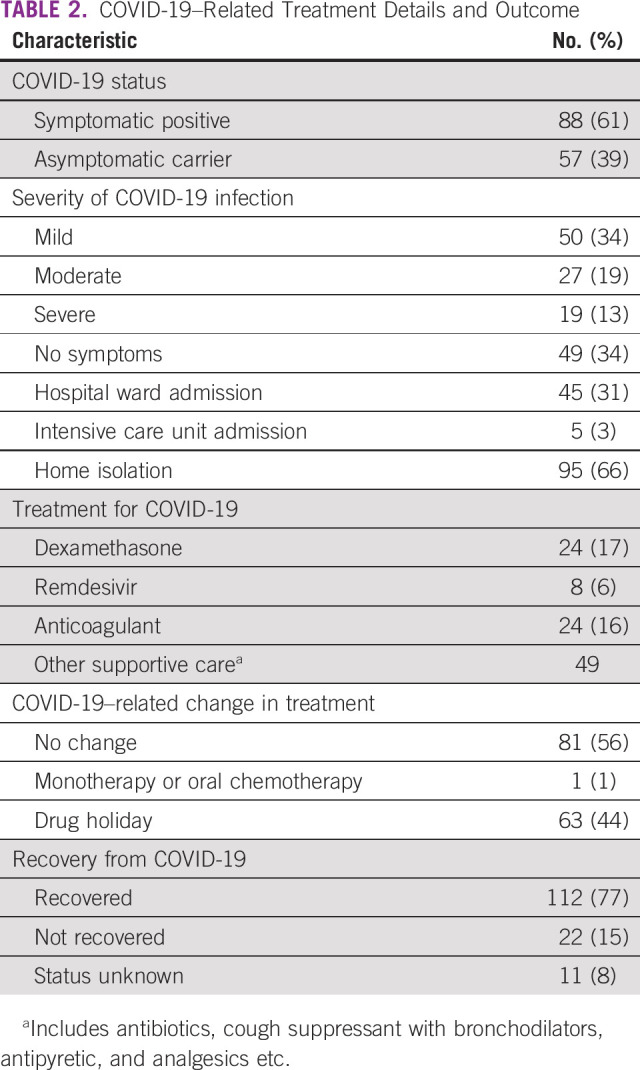
COVID-19–Related Treatment Details and Outcome

COVID-19–related death occurred in 22 (15%) patients, recovery in 112 (77%) patients, and status was unknown in 11 (8%) patients who never turned up after home isolation or treatment at other centers. COVID-19–related mortality was significantly associated with severity (moderate to severe) of symptoms (odds ratio, 91.31; 95% CI, 9.06 to 919.53; *P* = .0001). Elderly (age > 60 years), Eastern Cooperative Oncology Group performance status, presence of comorbidities, and intent of treatment (either curative or palliative) were not significantly associated with predicting mortality. The odds of death based on NLR was 0.99 (95% CI, 0.87 to 1.1; *P* = .94) and with PLR was 1.0 (95% CI, 0.99 to 1.00; *P* = .27). CFR was higher in lung cancer (36%) and genitourinary cancer (27%) compared with breast (13%) and gynecologic malignancy (4%). Factors predicting for mortality are summarized in Table 3.

**TABLE 3:**
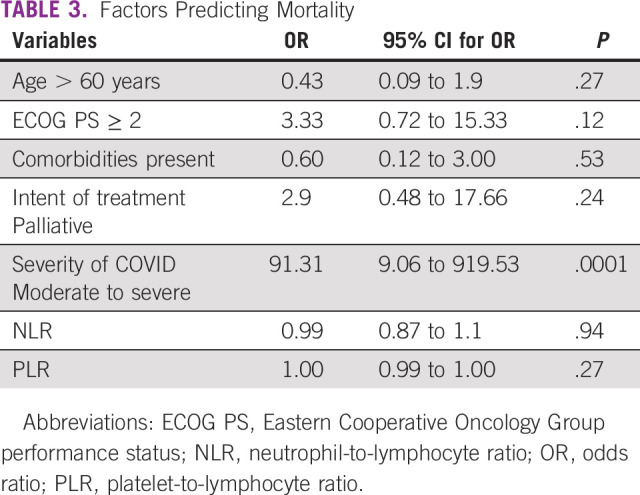
Factors Predicting Mortality

Those who did not require hospital admission were strictly advised for home isolation with a median duration of 25 days (range, 8-68 days) till the repeat test came negative. The median duration of test result negativity was 16 days (range, 7-62 days).

## DISCUSSION

The COVID-19 pandemic and nationwide lockdown created a significant negative impact on cancer treatment mainly because of major shifts in health resource allocation, medical facilities, and dedicated COVID hospitals, etc. Because of travel restriction, fear of infection, and various government guidelines, there was significant reduction in the number of patients availing cancer-directed services across the country. In our previous study, we had noticed that many patients were more worried about cancer progression rather than COVID-19 infection and despite lockdown and travel restriction, they were willing to continue systemic therapy.^10^ In this current study, we share our real-world experience of the outcome of COVID-19 cases on active systemic therapy over one year.

Several guidelines and expert recommendations were published regarding the approach to patients with cancer, especially how to deal with tumors of curative potentials while there is a delay in palliative systemic therapy for cases with questionable survival benefit.^11-13^ Our study also addresses how far these guidelines actually made an impact in the real world on the outcome of COVID-19–positive cases on systemic therapy.

The most prevalent cancer type in this study were lung followed by breast cancer, which is in contrast to the data from the UKCCMP but similar to some extent with the CCC-19 literature.^4,14^ The majority of patients had advanced metastatic diseases (62%) with palliative intent. Although drug holiday was given in 44% patients till the repeat test became negative, we continued the same treatment in the remaining 56% of population (those mainly on nonmyelosuppressive regimen, oral tyrosine kinase inhibitor, asymptomatic carrier, or mild symptoms with home isolation) along with COVID-19 infection.

According to Liang et al,^15^ CFR and morbidity among patients with cancer were higher than among noncancer patients, and those with significant comorbidities such as diabetes, hypertension, and ischemic heart diseases had a higher risk of mortality from COVID-19 infection. A meta-analysis of 32 studies involving 46,499 patients (among them, 1,776 patients with cancer) with COVID-19 across almost all countries showed that all causes of mortality and need for ICU admission were more common among patients with cancer; however, the elderly population with cancer may not be at increased risk of death.^16^

In our study, we identified that the majority (77%) were recovered from infection and the CFR was only 15%, markedly lesser than the mortality seen from the UKCCMP database (28%) and published US literature (25%-28%).^4,6^ The CFR in this study is quite similar to the data reported from Delhi, India (CFR, 14.52%), but higher than that from a report from Mumbai, India (CFR, 6.5%).^8,9^

We identified multiple possible factors that might explain the lower rates of mortality compared with Western data and other published reports. A majority (68%) of patients in this study had either no symptoms or mild symptoms. Second, the hematologic malignancies were not included as a part of this study, where reported CFR was maximum. Most of the studies so far reported their CFR data based on limited time-series information, which might be another cause of disparities. Finally, resource allocation toward more outpatient-based care, strict home isolation among asymptomatic positive or mild symptomatic cases, and racial disparities in addition to underlying biologic factors might be associated with a lesser rate of mortality from COVID-19 infection in this study.

As this pandemic continues, several prognostic markers, as well as inflammatory markers (such as D-dimer, lactate dehydrogenase, interleukin-6, and C-reactive protein), are now being identified to correlate with symptoms and diseases severity.^6^ Several studies reported that the NLR and PLR can be used as independent prognostic markers of disease severity in COVID-19.^17-19^ We identified that there was no significant association of NLR and PLR values in terms of mortality of our patients. We also observed that many of the other risk factors for mortality in this cancer cohort were comparable with published data from the various epicenters of this global pandemic. Our current study also suggests that patients with active systemic therapy take a median of 16 days to undergo test result negativity, although larger prospective data will be required to verify this finding.

Our study has its own strengths and limitations. Although retrospective in nature, this study provides comprehensive demographic profile of 145 COVID-19–positive cases with their treatment details and outcome among patients with solid organ malignancies. However, because of the limited follow-up period, it is difficult to draw conclusions by reading the late sequelae of recovered cases from COVID-19 infection. Apart from this, we were not able to correlate the value of D-dimer, lactate dehydrogenase, interleukin-6, and C-reactive protein with outcome, and inconclusive reports of RT-PCR were not included in the recovery list from COVID-19 infection. As testing was done only for those with symptoms, those who require admission, or planned elective procedure, there may be a chance of selection bias and under-reporting of actual COVID-19 prevalence of our patients.

In summary, the COVID-19–related CFR was 15% among patients with solid organ malignancies on active systemic therapy. Only the severity of COVID-19–related symptoms correlated with mortality. The majority of patients with mild symptoms were managed with home isolation and did not require further hospitalization. Drug holiday was given to 44% patients and 10% patients had progressive cancer thereafter. The systemic therapy of solid organ malignancies even in the COVID-19 era should be continued as per the existing standard of care. As the pandemic is ongoing, more prospective studies are needed to develop country-specific and cancer-specific guidelines, as well as strategies to treat patients with cancer with concurrent COVID-19 infection to overcome morbidity and mortality.
